# Animal Models of Diabetic Retinopathy: Summary and Comparison

**DOI:** 10.1155/2013/106594

**Published:** 2013-10-27

**Authors:** Angela Ka Wai Lai, Amy C. Y. Lo

**Affiliations:** ^1^Department of Ophthalmology, Li Ka Shing Faculty of Medicine, The University of Hong Kong, Hong Kong; ^2^Research Center of Heart, Brain, Hormone and Healthy Aging, Li Ka Shing Faculty of Medicine, The University of Hong Kong, Hong Kong

## Abstract

Diabetic retinopathy (DR) is a microvascular complication associated with chronic exposure to hyperglycemia and is a major cause of blindness worldwide. Although clinical assessment and retinal autopsy of diabetic patients provide information on the features and progression of DR, its underlying pathophysiological mechanism cannot be deduced. In order to have a better understanding of the development of DR at the molecular and cellular levels, a variety of animal models have been developed. They include pharmacological induction of hyperglycemia and spontaneous diabetic rodents as well as models of angiogenesis without diabetes (to compensate for the absence of proliferative DR symptoms). In this review, we summarize the existing protocols to induce diabetes using STZ. We also describe and compare the pathological presentations, in both morphological and functional aspects, of the currently available DR animal models. The advantages and disadvantages of using different animals, ranging from zebrafish, rodents to other higher-order mammals, are also discussed. Until now, there is no single model that displays all the clinical features of DR as seen in human. Yet, with the understanding of the pathological findings in these animal models, researchers can select the most suitable models for mechanistic studies or drug screening.

## 1. Introduction 

Diabetic retinopathy (DR) is a one of the most common microvascular complications of diabetes. In 2012, there are more than 371 million people suffering from diabetes, and it is being projected that the number of diabetic patients will reach 550 million in 2030 (http://www.eatlas.idf.org/; assessed 29-Nov-2012). Diabetes can be generally divided into two types: type 1 (insulin dependent) and type 2 (insulin independent), although patients of both types will have hyperglycemia. A study reported that about one-third of the diabetic patients have signs of DR and about one-tenth of them even have vision-threatening retinopathy [1]. Nearly 60% and 35% of DR patients progress to proliferative DR and severe vision loss in 10 years, respectively [2]. 

Clinically, DR can be classified into nonproliferative (NPDR) and proliferative (PDR) [3]. NPDR can be further graded into mild, moderate, and severe and is characterized by the presence of microaneurysms, hemorrhages, hard exudates (liquid deposits), cotton wool spots, intraretinal microvascular abnormalities, venous beading, and loop formation. NPDR may develop into PDR, where hallmarks of neovascularization of the retina and vitreous hemorrhage are found. Vision loss can be resulted from retinal detachment if patients are left untreated. Moreover, maculopathy, including macular edema and ischemia, can occur at any stage of DR; it accounts for the majority of the blindness due to DR. In fact, the growing number of diabetic patients and a longer life span in the aging population imply an increase in patients suffering from DR, which not only affects the quality of life of the individuals and their families but also increases the medical and economical burden to the society. As a consequence, effective therapy is urgently needed. 

In order to develop effective drugs, detailed understanding of the pathophysiological progression of DR is required. Over half a century ago, histological studies have been performed in postmortem retinas of diabetic patients. In retinal vessels and capillaries, selective endothelial and mural cells loss, presence of mural cell ghosts, endothelial clusters, acellularity, and microaneurysms were found to be increased in diabetic patients [4, 5]. Basement membrane thickening, presence of hemorrhage in the inner nuclear layer (INL), and outer plexiform layer (OPL) as well as eosinophilic exudates in the OPL were also reported [5].

Nowadays, immunological studies evidenced an increased glial fibrillary acidic protein (GFAP) expression in the Müller cell processes throughout the inner and outer diabetic retina, suggesting that these cells were hypertrophied [6]. There was also increased apoptosis in diabetic retina [7]. Abu El-Asrar et al. [8] further showed that proapoptotic molecules were expressed in ganglion cells, together with the activation of glial cells, which expressed several antiapoptotic molecules. Elevated vascular endothelial growth factor (VEGF) immunoreactivity was found in retinal blood vessels in diabetic humans with preproliferative or no retinopathy, further consolidated the role of VEGF in angiogenesis and vascular permeability [9]. Alternation in other factors, including somatostatin [10], cortistatin [11], *α*A and *α*B-crystallins, advanced glycation end products (AGEs), and receptor for AGE (RAGE) [12] as well as apolipoprotein A1 (ApoA1) [13], were also observed in the postmortem tissues. Somatostatin and cortistatin, which are neuropeptides with a very similar structure, were both downregulated in diabetic retinas, and their expression levels are inversely correlated with glial activation and apoptosis. On the other hand, upregulations of *α*A-, *α*B-crystallin, AGE, RAGE, and ApoA1 in the diabetic retinal tissue were reported. These morphological studies provide a better picture, yet without the mechanistic pathway, of the pathogenesis of DR at a cellular level. Moreover, advanced technologies further allow us to study the mRNA or protein expressions of various chemokines [14–17], cytokines [15–18], inflammatory markers [16, 19, 20], angiogenic factors [16, 17, 19–21], and other factors [19, 22, 23] in aqueous humor, serum, or urine from diabetic patients, thereby predicting the pathological pathways of DR. With the aid of the sophisticated computerized equipments and technologies, clinicians would even be able to monitor or predict the progression structural lesion [24–29] as well as functional defects [30, 31] in live patients with DR. 

Although a lot of important information or clues on the development of DR can be obtained from human studies, the mechanisms of DR development still cannot be elucidated. Emergence of animal models, therefore, not only enables us to have a more comprehensive understanding of the etiology of DR at a molecular level in a controlled manner, but also fulfills the need for drug screening tools. With these models, we may even be able to discover the early markers for DR in the body fluids from diabetic patients. This not only allows a faster and more convenient screening but also serves as an alarm for diabetic patients before the presence of cellular or functional lesion. Until now, many studies on the pathogenesis of DR have been carried out in animal models. A cascade of events, including oxidative stress, inflammation, protein kinase C (PKC) activation, accumulation of AGE and sorbitol, and upregulation of rennin-angiotensin system (RAS) and VEGF, contribute to retinal vascular endothelial dysfunction as a result of hyperglycemia [3]. Based on the mechanistic studies, drugs targeting different molecules in the cascade are being developed. In order to evaluate the effect of the drug properly, reliable and appropriate animal models are required. Throughout the years, many animal models of DR have been developed; however, none of them can mimic the entire pathophysiological progression as observed in human. While most animal models only show the early symptoms of DR, some show the late stage proliferative angiogenesis. Researchers have to select an appropriate model or models which can compensate each other in order to address their research questions. In this review, we focus on the animal models of DR that researchers have used, briefly describe how the models were generated, highlight the morphological and functional changes in the retina, and finally discuss the strengths and weaknesses of each model.

## 2. Animal Models

### 2.1. Mouse Models of DR

In general, mice have been routinely used in many *in vivo* studies since they are small in size and therefore easy to handle and inexpensive to house. They also have relatively short life span that allows a shorter experimental turnover time. Indeed, mechanistic studies of DR have been carried out extensively in mice as these models share similar symptoms of early DR as in human. More importantly, the availability of a collection of transgenic and knockout mice allows researchers to study the role of particular genes, which may even be cell type specific, in the development and pathophysiological progression of DR. There are three main types of mouse models to study DR; the first two involve mice with hyperglycemia development either via pharmacological induction or inbreeding of mice with endogenous mutation while the third type focuses on pathological angiogenesis found in transgenic animals or induced by experimental procedures, in mice without diabetes. 

#### 2.1.1. Pharmacologically Induced Mouse Models of DR

Type 1 diabetes can be induced in mice by injection of chemicals, including streptozotocin (STZ) and alloxan, both of which are toxic to and therefore destroy the *β*-cells in the pancreatic islets. Hyperglycemia can also be induced in mice by feeding with galactose. 

STZ-induced mice have been routinely used as a DR model in a lot of mechanistic studies and therapeutic drug testing for a long period of time owing to the abundant reports on the phenotypes. Depending on the injection dosage, the onset of diabetes can be achieved within a few days after injection in the wild-type animals, making it a popular diabetic model. Nevertheless, there are many variations in the injection protocol in terms of dosage, route of injection, and with or without insulin compensation that are usually based on the practice in individual laboratories; nonetheless, all mice end up with hyperglycemia in 1 to 4 weeks after STZ injection. For an easy reference, a summary of DR studies using STZ-induced mice in the past five years is presented in Table 1. This table aims to provide the researchers with a general idea of the appropriate dosage or injection method in mice of different age and/or with genetic background. Amongst these methods, intraperitoneal injection of single high-dose injection of 150 mg/kg and multiple low doses of 50 mg/kg for 5 consecutive days to C57Bl/6 mice without insulin compensation are the standard protocols recommended by the Animal Models of Diabetic Complications Consortium (http://www.diacomp.org/; accessed on 9-Dec-2012). In the STZ-induced diabetic mice, transient astrocyte activation [32] as well as increased astrocyte number [33] was observed at 4 to 5 weeks of hyperglycemia. GFAP upregulation in glial cells and reactive gliosis were also evidenced at the same time [33]. Retinal ganglion cells (RGCs) are reduced starting from 6 weeks [34, 35] while thinning of INL and ONL were observed at 10 weeks of hyperglycemia [35]. Apoptosis of RGCs and vascular cells can be identified at 6 weeks [35] and 6 months [32] of hyperglycemia, respectively. Yet, some studies showed that there is no significant in ganglion cell death even after a long time, up to 9 to 10 months of hyperglycemia [36–39]. Increased leukocyte number [40], together with leukostasis [38], was reported at 8 weeks of hyperglycemia. For vascular pathogenesis, upregulation of vascular permeability was being observed as early as 8 days of hyperglycemia [41], resulting in vessel leakage at 2 months [42]. After 17 weeks of hyperglycemia, thickening of capillary basal lamina [43] and neovascularization [44] were reported. Acellular capillaries and pericyte ghosts were found in retina in mice after 6 to 9 [32, 37] months of hyperglycemia. Decreased retinal arteriolar and venular RBC velocities, retinal arteriolar and venular blood flow rates, and arterial velocity were observed in mice after 4 weeks of hyperglycemia [45–47]. Although decreased arteriolar and venular diameters were also reported in mice after 4 weeks of hyperglycemia [48], it is controversial in other studies. Functional defects were described in some electroretinography (ERG) studies including decreased OP3 and ΣOPs, prolonged implicit time of OP2-3 at 4 weeks of hyperglycemia [49, 50]; decreased a-waves and b-waves at 6 months of hyperglycemia [37]; and decreased pattern ERG amplitude at 7 weeks of hyperglycemia [51]. The variations in the above observations may be due to difference in mouse strains [52], STZ dosage, or observation time points. Moreover, individual animals may be resistant to STZ and fail in hyperglycemia induction; therefore, it is essential for the experimenters to confirm the blood glucose level of the animals and exclude those without hyperglycemia development.

On the other hand, alloxan is less commonly used in mice, which may be due to the absence of the inducible cellular and vascular lesions associated with hyperglycemia. Morphologically, the dendrites of microglial cells were found to be shortened without any ganglion cell apoptosis after 3 months of hyperglycemia [128]. At the same time, vessel diffusion and density of the retinal capillary network remained unchanged [128]. Functional abnormalities, however, were being reported in ERG, in which b-wave amplitude [129, 130] and b/a wave amplitude ratio [128] were found to be decreased at 3 weeks and 3 months of hyperglycemia, respectively. Nevertheless, cellular and vascular lesions are possibly detectable after a longer period of hyperglycemia as suggested by the presence of functional defects in this model.

Feeding with galactose is another method to induce hyperglycemia. Here, the blood aldohexose concentration is elevated in the animal without affecting other metabolic abnormalities, such as alterations in concentrations of insulin, glucose, fatty acids, and amino acids. This allows the researchers to study the consequential retinal complications solely due to the elevation of hexose concentration. Galactose-fed mice have a longer life span than other diabetic models; therefore, an extended monitoring period of up to 26 months can be allowed [131–133]. Endothelial cell loss and increased acellular capillaries were observed starting from 15 months of hyperglycemia. For a further 6 months of hyperglycemia, other morphological lesions including the presence of pericyte ghosts, saccular microaneurysms as well as basement membrane thickening of retinal capillaries were also evidenced [131–133]. Amongst the mouse models currently available, the animals in this model have the least mortality at around 2 years old of age, which allows a longer period of hyperglycemia, and therefore phenotypes associated with increment of hexose concentration can be targeted. Researchers should be aware that it takes a relatively longer period of time to develop retinopathy in these mice, which in turn leads to a higher cost.

#### 2.1.2. Diabetic Mouse Models Carrying Endogenous Mutation

Apart from injection or intake of chemicals, spontaneous hyperglycemia can be found in mice carrying endogenous mutation. By inbreeding the mutated mice with the wild-type animals, researchers can further expand the colonies and use them as mouse models for diabetes studies. Although breeding is a time-consuming process, further manipulations, such as injections and feeding with specialized chow, are avoided. Retinopathies in terms of morphological and functional lesions have been observed in a few type 1 and type 2 diabetic models, including Ins2^Akita^, nonobese diabetic (NOD), db/db, and KKA^y^ mice. In these animals, onset of hyperglycemia takes place spontaneously as a result of the presence of the transgene or mutation; a relatively consistent phenotype as well as a higher success rate in induction of hyperglycemia can be obtained. 

The Ins2^Akita^ mice represent one of the spontaneous type 1 diabetic models. These mice carry a point mutation in the insulin2 gene, which causes a conformational change in the protein that accumulates in the pancreatic *β*-cells, and ultimately leads to cell death. Retinopathy in heterozygous Ins2^Akita^ mice is well characterized in a few studies. Cellular lesions, such as reactive microglia, are evidenced as early as 8 weeks of diabetes [134]. By 12 weeks of onset of hyperglycemia, immunological studies showed abnormal swelling in somas, axons, and dendrites of RGCs, and the number of these cells was reduced in the peripheral region; while more dendritic terminals, increased total dendrite length, and greater dendritic density were observed in the ON-type RGCs [135]. It has been reported that the number of RGCs was significantly reduced in the peripheral regions after 22 weeks of hyperglycemia [135]; yet, another study showed that there was no ganglion cell death in these mice even up to 10 months of hyperglycemia [39]. Morphological change in astrocytes was also observed where they had short projections and became in less contact with the vessels [134]. Moreover, the IPL and INL became thinner, which may be due to the decrease of the cholinergic and dopaminergic amacrine cells as evidenced in retina after 6 months of hyperglycemia [136]. Vascular lesions such as increased of leukocyte number are already found in mice upon 8 weeks of hyperglycemia and retinal vascular permeability was increased in 12 weeks of hyperglycemia. The presence of acellular capillaries [134] and neovascularization [137] were described after 8 to 9 months of hyperglycemia. Abnormal vascular functions were also reported in a study which showed that the arteriolar and venular RBC velocity, shear rate, and retinal blood flow rate were significantly decreased in the diabetic animals with 26 weeks of hyperglycemia [138]. A decrease of a- and b-wave amplitudes in the ERG after 8 month indicates functional problem associated with the cellular defects or degeneration [137]. Although the diabetic animals have an average life span of 305 days, they provide a stable induction of hyperglycemia while projecting the early and some of the late DR symptoms in human. Thus, this model could be very useful in drug screening, and it has received more attention in the field of DR.

The NOD mice are another model of type 1 diabetes, in which pancreatic *β*-cells were destroyed via an autoimmune process by the CD4^+^ and CD8^+^ cells. The onset of hyperglycemia in these animals is 12 weeks of age, and the frequency of having hyperglycemia at the age of 30 weeks old is about 80% in female and less than 20% in male [139]. Owing to the low induction rate and inconsistency in the male mice, female NOD mice were commonly used. Using transmission electron microscopy, ultrastructural changes including apoptosis of pericytes, endothelial cells, and RGCs, perivascular edema, and retinal capillary basement membrane thickening were reported as early as 4 weeks of hyperglycemia, and these retinopathogeneses became more obvious after 12 weeks of hyperglycemia [140]. At about 4 months of hyperglycemia, vascular abnormalities were described in the NOD mice. Vasoconstriction or degeneration was observed in some of the major vessels, together with the presence of poorly defined microvessels and disordered focal proliferation of the new vessels [141]. The presence of these vascular pathologies and the etiology of the development of type 1 diabetes in the NOD mice are relatively similar to those of human, making this model unique. However, there is a big variation in the time of onset of diabetes; frequent and regular monitoring of the blood glucose levels is therefore required. More importantly, since female animals were used in these studies, estrogen, which plays a role in regulation of metabolism [142], may have a protective function in DR. This further complicates the mechanistic studies or contributes unknown effects in drug screening, thereby affecting the accuracy.

The db/db mice spontaneously develop type 2 diabetes owing to the deficiency in the leptin receptor. Hyperglycemia and obesity were observed in the homozygous mice at 4 to 8 weeks old [143]. Reduction of RGC number and thickness of the central total retina, INL, and photoreceptor layers were identified in the histological sections of mice after 6 weeks of hyperglycemia [144]. After 18 weeks [145] and 13 months of onset of hyperglycemia [146], pericyte loss and glial reactivation were reported, respectively. Vascular lesions including capillary basement membrane thickening [147], the presence of acellular capillaries [145], increased vessel density in the INL, and vessel leakage [146] were observed in the diabetic retina. Compared with other mouse models, the reported glial reactivation and vessel leakage occurred relatively late in db/db mice, which could be explained by a relative late time point chosen in a long-term study [146]. The use of db/db mice to study DR is not very popular, potentially due to a low birth rate resulting from the unsatisfactory mating performance in the male homozygotes and failure to reproduce in the female homozygotes (http://jaxmice.jax.org/; accessed on 15-Dec-2012).

KKA^y^ mouse is a combined model made by the introduction of the yellow obese gene, A^y^, into the KK mouse, in which moderate diabetic traits are thought to be inherited by polygenes. These mice spontaneously develop diabetic characteristics, such as hyperglycemia, hyperinsulinemia, and obesity, at around 6 to 8 weeks of age, but revert to normal at the age of 40 weeks [148]. Of the limited DR studies using this mouse model, increased apoptosis of the retinal neuronal cells was found in the RGC layer and the INL in mice after 4 weeks and 3 months of hyperglycemia, respectively. Capillary basement membrane thickening was also observed in ultrastructural study [149]. Owing to the limited retinopathologic findings and the uncertain etiology of this model, this model is not popularly used in DR studies.

#### 2.1.3. Mouse Models of Proliferative Retinopathy

In order to compensate for the lack of proliferative pathogenesis in the retinal vasculature in most of the above diabetic mouse models, researchers developed a number of nondiabetic models, which allow them to specifically target neovascularization in the ocular region. When using these models, however, researchers should be aware of the fact that the etiology of the progression of vascular abnormalities is different owing to the absence of classical systemic characteristics as seen in diabetes. Proliferative retinopathy can be achieved mainly from two approaches: the first one is via introduction of ischemia to the eyes, such as oxygen-induced retinopathy (OIR) and retinal occlusion [150]; the second one is by direct injection or genetically induction of the angiogenic factor, VEGF, into the ocular region. 

OIR originally is an animal model of retinopathy of prematurity in newborns. Owing to the presence of neovascularization, this model is also adapted for studying the angiogenesis as seen in proliferative DR [150]. In brief, postnatal day 7 (P7) neonatal mice were put into a 75% oxygen chamber for five days and then returned to room air [151, 152]. Vessel loss in the central area of retina, which is associated with hypoxic challenged, is observed immediately at this time. Neovascularization extending from the inner retina into the vitreous begins at two days after the return to room air, peaks on P17 [152], and is gradually regressed and spontaneously resolved by P25 [151]. A comprehensive study was carried out in this model, in which mice at P18 were analyzed [153]. Morphologically, more gliotic Müller cells and reactivated microglia can be observed. The total retinal thickness was reduced in the midperipheral region, while the IPL and the outer segment length were reduced in the central and midperipheral regions. The number of vessels was also reduced in the IPL in the central region; and in the deep plexus in the central and midperipheral regions. Aberrant intravitreal neovascularization was observed across all eccentricities. Retinal function was examined by ERG, in which reductions in the amplitudes of a-wave, b-wave, OP3, and OP4, and delayed b-wave implicit time were revealed. Mouse OIR model showed a number of vascular, neuronal, and glial changes in the retina; however, the spontaneous regression of the neovascularization within a week confines its application in therapeutic drug research.

Since DR can also be considered as an ischemic disorder in the retina, retinal occlusion models, such as unilateral ligation of pterygopalatine artery (PPA) and external carotid artery (ECA) [154], branch retinal vein occlusion [155], and elevating the intraocular pressure (IOP) [156], were also applied in studies of vasculature abnormalities. In these occlusion models, increased apoptotic cells, reduced thickness of retinal cell layers, reduced a-wave, b-wave, and OPs amplitudes of the ERG were evidenced. Nevertheless, the acute induction of ischemia, particularly those followed by reperfusion, to the retinal tissue makes these models less appropriate for studying DR, in which chronic ischemia is persistently involved.

Kimba (trVEGF029) is a transgenic mouse model of neovascularization, as a result of transient overexpression of human VEGF_165_ in the rhodopsin-expressing cells with which it peaks at P10 to P15 and declines at P20. Characterization studies have been carried out as early as at P7 [157]. At that time, reduced thickness of the RGC layer, INL, outer nuclear layer (ONL), and the total retinal layer was observed. By P28, such reduction was found in IPL, the outer segment, and the total retinal layer. Microaneurysms, intraretinal microvascular abnormalities, and capillary dropout were evident by P28 [157]. Vascular leakage was also observed at P28 [157], but it ceased at 9 weeks of age potentially due to the absence of over-expression of VEGF [158]. Increased adhered leukocytes were found in veins and capillaries, together with increased acellular capillaries by 6 weeks of age. Topological and fractal analysis of retinal vasculature showed that vessel-covered area, vessel length, and crossing points in the 9-week-old Kimba mice were reduced. Vessel tortuosity and pericyte loss were also evident at this time point [158]. Varied degrees of the pathogenesis were being reported, which were separated into two groups based on the assessment of fundus fluorescein angiography. The phenotypic observations of the relatively severe group were presented in this review. Since this transgenic mouse is not commercially available, it is not a popular model in DR.

In order to generate an ideal mouse model to study DR, a new mouse model of DR was created by crossing the Kimba mice with the Ins2^Akita^ mice, named Akimba [159]. These mice inherit the key systemic diabetic phenotypes from their parental strains, making it a unique model. At 8 weeks of age, optical coherence tomography (OCT) showed uneven thickness in the retina. Retinal edema and reduced photoreceptor layer thickness, together with retinal detachment, were observed. RGCs number and neural retinal thickness were reduced by 24 weeks of age. Abnormal microvasculature, including microaneurysms, capillary dropout, hemorrhage, neovascularization, venous loops, vessel tortuosity, vessel beading, vascular dilatation, and vascular leakage, were also evident in mice of 8 weeks old, although the leakage stopped by 20 weeks of age. The Akimba mouse model displays a number of vascular changes; however, more complete mechanistic studies are essential before its utilization in therapeutic drug studies. 

A summary of the earliest reported morphological and functional lesions in different mouse models of DR is shown in Table 2.

### 2.2. Rat Models of DR

Although rats have a slightly bigger size than mice, they are still easy to handle with a low cost of maintenance, making them to be another popular animal frequently used in *in vivo* studies. The use of rats in DR study is particularly common owing to a relatively larger tissue size, with which functional assessment and morphological and molecular analyses can be done. Similar to DR studies in mice, three types of rat models were used; these include pharmacological induction of hyperglycemia, spontaneous diabetic rats, and models of angiogenesis without diabetes. A summary of the morphological and functional lesions in different rat models of DR is shown in Table 3.

#### 2.2.1. Pharmacologically Induced Rat Models of DR

Similar to mice, hyperglycemia can be induced in rats by injection of STZ or alloxan or by ingestion of galactose. 

Compared to mice, rats are more susceptible to the toxicity of STZ; therefore, usually a much lower dosage of STZ is used. In order to minimize the mortality, insulin complementation may also be administered. Table 1 summarized most of the induction method of STZ in the past 3 years and served as a reference for the researchers to select the most appropriate dosage, injection paradigm, and rats with different genetic background for their studies. Amongst the methods, a single dose of 60–65 mg/kg of weight is the most popular one. Variation of the retinal lesions was reported, which can be explained by the genetic background. Indeed, a comparative study showed a strain difference in the rate of developing early DR symptoms in rats upon STZ challenge [120]. After 8 months of hyperglycemia, Lewis rats displayed accelerated degeneration of retinal capillaries and RGC loss, whereas Wistar rats only showed the capillary degeneration and Sprague-Dawley (SD) rats showed no morphological defects to a significant level. Increased apoptotic cells were detected throughout the retina as soon as 2 weeks after hyperglycemia [136]. After 1 month of hyperglycemia, the number of astrocytes in the peripheral region was reduced; however, the number of Müller cells and microglial cells is increased [160, 161] with the accompany of microglial hypertrophy [160]. The density of astrocyte was further reduced in the central region, together with the reduction of astrocyte processes in the peripheral region after 6 weeks of hyperglycemia [118]. Decreased total retinal thickness [163], as well as decreased number of cells in the RGC layer [160], the ONL [163], and the INL [160] was also reported. Regarding the vascular changes, blood-retinal-barrier (BRB) breakdown was evident at 2 weeks of hyperglycemia [161, 163]. Increased adherent leukocytes [164] and arterial or venous capillaries basement membrane thickening [165] were observed at 8 weeks and 12 months after hyperglycemia, respectively. Retinal function was affected from 2 weeks after the onset of hyperglycemia as reflected by the ERG. Reduced b-wave [166], OPs [167], and a-wave [166] amplitudes were progressively found at 2 weeks, 8 weeks, and 10 weeks of hyperglycemia. At around the same time, the a-wave implicit time and OPs were delayed [168]. Morphological and functional studies suggested that STZ-induced diabetic rats only showed early DR symptoms, which is comparable to those in STZ-induced mice.

The use of alloxan in DR studies is not very common nowadays, and the existing morphological studies were mainly focused on the vascular lesions. Neovascularization was already observed from 2 months of induction of hyperglycemia starting from the midperiphery region and progressing to the whole retina after 9 months of hyperglycemia [170]. Extravascular macrophage accumulation and capillary endothelial cells swelling were also identified after 2 months and 5 months of hyperglycemia, respectively [170]. By 8 months, increased cell death in the retinal microvasculature was evident [171]. Acellular capillaries, basement membrane thickening, and pericyte loss were also reported upon 12 months of hyperglycemia [169]. Nevertheless, appearance of the lesions varied between studies; it would be due to the different time points selected by the authors or different dosage of alloxan being injected. Reported lesions with the “earliest onset” were listed in this review. 

Similar to mice, DR can be studied in rats fed with galactose, with the equivalent advantage of longer life span [172, 198]. Studies showed increased retinal microvascular cell death in 6 months after hyperglycemia [171]. Other vascular lesions, such as acellular capillaries and basement membrane thickening as well as pericyte loss, were observed after 12 months of feeding with galactose [169]. A long-term study demonstrated cellular lesions, including gliosis and disruption of retinal layers, together with vascular abnormalities, capillary dilation and microaneurysm formation in the inner plexiform layer (IPL) and the INL, in rats fed with galactose for 28 months [172]. Owing to the differences in galactose concentration and time points selected by various studies, the “earliest onsets” of the lesions were listed in this review.

#### 2.2.2. Diabetic Rat Models Carrying Endogenous Mutation

A number of rats with spontaneous onset of diabetes have been identified. These include type 1 diabetic model: biobreeding (BB) as well as type 2 diabetic model: Wistar Bonn/Kobori (WBN/Kob) rats, Zucker diabetic fatty (ZDF) rats, Otsuka Long-Evans Tokushima fatty (OLETF) rats, nonobese Goto-Kakizaki (GK) rats, and nonobese spontaneously diabetic Torii (SDT) rats.

Like NOD mice, the BB rats spontaneously develop polygenic autoimmune type 1 diabetes, in which the pancreatic *β*-cells were selective destroyed [175, 199]. After 4 months of hyperglycemia, absence of infolding and derangement of the basal plasma lemma of the RPE were also observed [173]. The number of pericyte and the pericyte to endothelial cell ratio were reduced [174, 175] after 8 months of hyperglycemia. Lesions associated with the retinal microvasculature, including capillary dilation and basement membrane thickening, were found from 2 months and 4 months of hyperglycemia, respectively [175]. Microinfarctions with areas of nonperfusion were evident whereas no neovascularization was detected up to 11 months [175]. Several inbred and outbred lines, such as BB/Wor, BB/E, and BB/Ph, have been produced and named based on the origin of the breeding colony. Since genetic variations and potential differential phenotypes are introduced, researchers should select the appropriate stain carefully.

The WBN/Kob rats are a type 2 diabetes model owing to endo-exocrine pancreatic insufficiency, and only male offspring develop diabetic symptoms [200]. Retinal degeneration was already observed before the animal becomes hyperglycemic at around 9 to 12 months of age. Thickness of outer segments and ONL was reduced in WBN/Kob rats at 5 months of age whereas high blood glucose was evident in these rats at 10 months old [176]. About 2 months after becoming hyperglycemic, these rats also showed reduction in the visual cells, the OPL, and the total retinal layer [176]. Vascular lesions were also identified, and capillaries clustered into small tortuous knots after about 1 month of hyperglycemia [177]. After 5 to 6 months of diabetes, capillary basement membrane thickening [176], increased capillary loop, and reduced number of capillary [177] were also observed. After a prolonged hyperglycemia of 12 months, some rats showed increased proliferation of fibrovascular element in the vitreous, intraretinal neovascularization, and hyalinization of intraretinal vessels [178]. WBN/Kob rats, which display the symptoms of the progressive DR, may serve as a model for testing therapeutic drug targeting angiogenesis. However, the early onset of neuronal degeneration (before hyperglycemia commencement) suggests that the etiology of retinal degeneration may not be the same as that in human; therefore, further studies need to be carried out before this can be used as a model for DR.

The ZDF rats are also a genetic model of type 2 diabetes. They carry an inherited obesity gene mutation, which results in impairment of glucose tolerance and insulin resistance (http://www.criver.com/SiteCollectionDocuments/ZDF.pdf; accessed on 19-Nov-2012). Excessive body weight gain was observed in male ZDF rats in the first 6 months of life, but the weight decrease to a level similar to the lean controls afterwards [201]. Hyperglycemia starts at 6 to 7 weeks of age and maintains high throughout their life [180]. Retinopathological studies in these rats mainly focused on the vasculature. Thickening of the capillary basement membrane and increased capillary cell nuclear density were reported in rats after 5 months of hyperglycemia [180, 181]. Apoptosis of endothelial cells and pericytes was higher in these rats compared to the lean controls, together with an increased number of acellular capillaries and pericyte ghosts upon 6 months of diabetes [179]. Retinal functional analysis and long-term morphological studies of the retinal neuronal and glial cells of these rats remain to be elucidated.

Another type 2 diabetic rat model is OLETF rats; they significantly gained more weight from 1 to 6 months of age, but they lost weight from 9 to 10 months of age. Elevated blood glucose was observed from 5 months of age and it maintained high [182, 183]. After about 6 months of hyperglycemia, despite no significant difference in the number of acellular capillaries and pericyte ghosts [202], OLETF rats with 9 months of hyperglycemia showed reduced ratio of pericyte area to the total capillary cross-sectional area [183] and damaged endothelial cells [182]. By 14 months of hyperglycemia, the INL and the photoreceptor layer became thinner, accompanied with shortening of the RPE height and poorly developed basal infoldings [182]. Relatively early microvessel-related symptoms were reported in these rats, in which leukocyte entrapment was evident in rats after 6 weeks of hyperglycemia [184]. Other abnormalities, including thickening of capillary basement membrane, tortuosity, microaneurysms, loop formation in capillary, caliber irregularity and narrowing of arteries, were also described in rats after 9 to 12 months of diabetes [182, 183, 185]. No hemorrhages, emboli, and exudates were found in these rats up to 14 months of diabetes [182]. Moreover, ERG revealed that OLETF rats fed with sucrose for 8 weeks had a prolonged peak latency of ∑OPs [203]. The absence of acellular capillaries as well as the late onset of diabetes and the related symptoms diminished the popularity of using this model to study DR.

The GK rat is a spontaneous model of non-insulin-dependent diabetes without obesity. These rats are originated from normal Wistar rats and they were selected via repeated inbreeding exercise using glucose intolerance as a selection index [186, 204]. Rats at 4–6 weeks of age develop hyperglycemia [186, 187]; they also showed reduction of retinal segmental blood flows and prolonged retinal mean circulation time after 1 month of hyperglycemia [186]. Increased BRB permeability [187] and endothelial/pericyte ratio [186] were also evident in 3 months and 7 months after the onset of hyperglycemia. No significant differences were observed in the retinal arterial and venous diameters [186]. Owing to the limited publications on the retinopathology in the GK rats, further characterizations on the non-vascular-related lesions need to be performed.

The SDT rat, which is a substrain of the SD rat, is another model of nonobese type 2 diabetes. Glucose intolerance and impaired insulin secretion were demonstrated in the male SDT rats at 14 weeks, followed by hyperglycemia and glucosuria at 5 months of age. These rats showed a sexual differentiation in the development of diabetes that the cumulative incident is about 100% in males at 40 weeks of age and only about 33% in females up to 65 weeks of age [190, 205]. Retinal dysfunction was observed after 4 weeks of hyperglycemia, as evident by delayed peak latency of the ∑OPs [189]. The amplitudes of a-wave, b-wave, and ∑OPs were significantly reduced with prolonged implicit times at 24 weeks of hyperglycemia [193]. At the same time, leukostasis [192] and the number of apoptotic cells in the GCL and the INL [188] were increased in the retinas of SDT rats. Vascular lesions, such as acellular capillaries and pericyte loss, have been described by Kakehashi et al. [206]. Advanced lesions, including leakage of fluorescein around the optic disc as well as distortion of retina and protruded optic disc, were also observed after 48 weeks of hyperglycemia [189]. More importantly, a few studies showed that proliferative DR can be detected in some of the aged SDT rats, which have been exposed to hyperglycemia for more than 48 weeks [190, 191, 205, 206]. The reported symptoms include retinal hemorrhages, tortuous vessels, capillary nonperfusion, neovascularization, and tractional retinal detachment with fibrous proliferation. Amongst the diabetic rat models mentioned in this review, the SDT rat is the only one that shows severe ocular complications similar to those seen in human. Although some common phenotypes in human DR, such as microaneurysms and development of avascular area, are rare in this model, this is a unique model to study proliferative DR [205, 207].

#### 2.2.3. Rat Models for Angiogenesis Study

Similar to the mouse models for studying angiogenesis, OIR and occlusion models were also applicable to rats. Owing to the relevance of ischemic-induced neovascularization, we will focus on the OIR in this section.

The basic principle of OIR in rats is very similar to that in mice, which involves the induction of neurovascularization in nondiabetic animals. Different from the “standard” protocol of OIR in mice, several paradigms with varied oxygen concentrations and duration of the exposure period have been applied in rats. In brief, the newborn pups are exposed in alternative hyperoxia-hypoxia cycles for 11 to 14 days and then returned to room air [194, 195, 208–210]. Peripheral astrocyte degeneration was observed soon after the rats were exposed to room air [195]. At P18, the number of astrocyte was reduced almost throughout the whole retina [195] with prominent Müller cell reactivity in the regions that are devoid of intraretinal blood vessels [194]. Reduction of thickness in the INL, the IPL, and the total retinal layers was evident [194]; the outer segment layer also became thinner and disorganized [196]. While the number of pericytes was comparable to the room air control, the pericyte-endothelial interactions were impaired [195]. Intravitreal neovascularization, incomplete development of the outer vascular plexus and extension of the abnormal endothelial “tufts” toward the vitreous were observed in the OIR rats [195]. Functional lesions were also studied using ERG, in which the a- and b-wave amplitudes were reduced [197]. This model is useful in therapeutic drug screening or in the study of the mechanisms in angiogenesis, yet special equipments are required. Moreover, strain-dependent difference in the degree of retinal vascularization and abnormalities in vascular morphology have been reported [208]. The albino SD, the pigmented Dark Agouti, and Hooded Wister rats were more prone to the hyperoxia-hypoxia challenge, and they showed severe vascular attenuation following the oxygen exposure as well as severe vascular pathologies when compared to other strains.

In summary, rodents are very popular models to study the pathogenesis and examine the efficiency of therapeutic drugs of DR in laboratories. They have the advantage of being small in size which allows easier handling; however, this also makes *in vivo* examinations, such as fundus photography, fundus fluorescein angiography, and optical coherence tomography, difficult. Despite the lack of proliferative DR symptoms as described in most of the models mentioned above, researchers also focus on other animals in order to obtain the most representative model of DR, which ideally displays the comparable DR symptoms as seen in human patients. Higher mammals not only can serve as a platform for easier examinations but also allow easier treatment particularly those involving sophisticated surgical procedures. In these animals, sampling of body fluid, for example, vitreous and blood, can also be performed routinely.

### 2.3. Rabbit Models of DR

Similar approaches have been applied to rabbits to induce DR; these include pharmacologically induced and dietary-induced diabetic models as well as VEGF-induced angiogenesis in the retina without affecting the blood glucose level.

#### 2.3.1. Pharmacologically Induced Rabbit Models of DR

Hyperglycemia can be induced in rabbit by STZ, although this method is not very frequently used. A study showed that intravenous injection of STZ (100 mg/kg) in rabbits can elevate their blood glucose level [211]. Fundus examination was done after 19 weeks of hyperglycemia and all eyes showed certain degree of retinopathy, of which 50% showed proliferant retinopathy; 40% showed serious vasculopathy with serious retinal and preretinal hemorrhages, vascular lesions, hemovitreous and venous thrombosis; and the remaining 10% showed moderate vasculopathy with hard or soft exudates and widespread hemorrhages. Variation in the extent of the retinopathogenesis limits the use of this model.

#### 2.3.2. Diet-Induced Rabbit Models of DR

Early DR can be found in rabbit models of diet-induced impaired glucose tolerance plus hyperlipidaemia [212]. Rabbits were fed with standard chow with 10% lard, 40% sucrose, and 0.1–0.5% cholesterol for a period of 24 weeks. The blood glucose level slightly elevated in the animals after feeding with 12 weeks of the special diet, and they became hyperglycemic by the end of the study period. Histological findings suggested that increased microaneurysms and hyperfluorescent dots were already present before the rabbit becomes hyperglycemic, while those pathological symptoms further progressed with prolonged feeding. Although this model mimics the natural development of type 2 diabetes in human, the drawback is the slow progression of DR symptoms.

#### 2.3.3. Rabbit Models for Angiogenesis Study

In brief, a polymeric pellet containing human recombinant VEGF was implanted into the vitreous cavity of the rabbit [213]. After 7 days of implementation, increased dilation and tortuosity of retinal vessels were observed. During 14 to 21 days after implementation, fluorescein angiography further showed profuse leakage of dye, together with the presence of numerous small tortuous blood vessels, suggesting induction of neovascularization. However, such vascular changes stopped afterwards and neovascularization almost totally regressed after 35 days of implementation. The authors suggested that the regression of vessels may be due to depletion of the VEGF, implying that choosing the experimental endpoint is crucial when screening therapeutic agents in this model. Therefore, another group generated a similar model, in which human recombinant basic fibroblast growth factor (bFGF) was also incorporated into the polymeric pellet besides the human recombinant VEGF [214]. In this model, similar retinopathologies were observed but they only required approximately half of the time to develop when compared with the VEGF-induced model previously described. In addition, hemorrhage from the new vessels and even total traction retinal detachment were also observed. Moreover, differential retinal angiogenic response to VEGF/bFGF was reported in different rabbit strains, where Dutch belt rabbits are more susceptible than the NZW/Black satin cross rabbit [215].

It is evident that vascular retinopathy could be observed in the rabbit models mentioned above; however, researchers should be aware of the fact that retinal vasculature in the rabbit differs from those in other species. In rabbit, the optic artery branches into major blood vessels in a bidirectional horizontal manner; they further arborized into capillaries, forming a ring-like network. Moreover, the visual streak of rabbit is located below this region; functional defects may not be able to be detected if the lesion site is in the medullary ray where the blood vessels are. As compared with other animals, such vascular system is only present in a small area of the retina in rabbit; therefore, the global deleterious contributions by the vessels may be underestimated. On the other hand, if researchers aim to study the vessel-to-cell interaction at a molecular level, this model provides an alternative choice with an additional advantage of a bigger eyeball size than rodents. Therefore, more precise and delicate experiments can be performed, but the problem of limited housing space needs to be attended to.

### 2.4. Cat Model of DR

The majority of the DR studies in cats are induced by pancreatectomy with or without alloxan injection. The animal will become hyperglycemic 1 to 2 weeks after the surgery [216–218]. Capillary basement membrane thickening was first described from 3 months of pancreatectomy, where no change was observed in the number of endothelial cells and pericytes as well as the contacts between endothelial cells and pericytes up to 10 months [218]. A case report showed that microaneurysm was first observed in one eye in a diabetic cat after 5 years of pancreatectomy; and by 6.5 years, both eyes showed microaneurysms and small intraretinal hemorrhages in the area centralis [216]. Region of capillary nonperfusion and intraretinal microvascular abnormalities (IRMA) were also evident from 7.5 years. At 8.5 years, presence of small foci of neovascularization was suggested. Cotton-wool spots, venous beading, extensive preretinal neovascularization, or microvascular changes were not found in the peripheral retina. On the other hand, another study showed that only one out of two experimental diabetic cats showed microaneurysms, but not hemorrhages or area of nonperfusion, after 7 years of pancreatectomy, while the other diabetic cat did not show any microaneurysm or hemorrhage in the retina [217]. Cats only showed mild cataracts upon diabetes, thereby allowing visualization of the fundus angiography and ERG. However, the studies of DR in cat are very limited and the described phenotypes are less consistent. A long follow-up period for the development of retinal pathology and lack of reagents in molecular studies may be the drawbacks for using this animal model.

### 2.5. Dog Model of DR

Attempts of using dogs for studying DR have also been made, in which most of them are about inducible hyperglycemia either by injection of STZ or alloxan or feeding the animals with galactose. It has been suggested that galactose-fed dog is the animal model that shares the retinal lesions morphologically and clinically as those developed in human diabetic patients [219].

#### 2.5.1. Pharmacologically Induced Dog Models of DR

Induction of diabetes in dogs by intravenous injection of STZ and alloxan resulted in basement membrane thickening in 3 years of injection, and it was recognized in some vessels from the first year [220]. Loss of pericytes and smooth muscle cells was observed in the retinal arterioles from 4 years of postinjection; no microaneurysm was noted towards the end of this 5-year study. Moreover, a comparative study showed that increased microaneurysms, acellular capillaries, pericyte ghosts, endothelial cells to pericytes ratio, and basement membrane thickening were evident in the dogs after 5 years of galactosemia than those of alloxan-induced diabetes [221].

In the galactose-induced DR model, experimental dogs were continuously fed with normal diet supplemented with 30% galactose. Cellular lesions such as presence of pericyte ghosts and uneven distribution of endothelial cells were observed in retina of dogs after feeding with galactose for 19 and 24 months, respectively, followed by the formation of microaneurysms [222, 223]. Dot and blot hemorrhages were found from 33 months, which became more confluent, progressing to the preretinal and intravitreal regions after 66 months of feeding [222]. Nonperfusion was evident in dogs from 37 months of feeding [223, 224], and the area was broadened with time [222]. After 36-month feeding of galactose, acellular capillaries and endothelial cells to pericytes ratio were increased [225]. Other vascular lesions, such as abnormalities in intraretinal microvessels, occlusion of arterioles, presence of large arteriovenous shunts, and node formation on arterial and arteriolar walls, were also reported after feeding for about 5 years. At about the same time, presence of soft exudates and gliosis in the nerve fiber layer was reported [222]. Further advanced retinopathy of neovascularization was described in dogs being galactose fed for 68 to 84 months [222, 224]. It has been suggested that the onset of DR symptoms is age dependent in galactose-fed dogs; younger animals develop DR symptoms earlier than the older ones [225]. This may explain the variation of the onsets of the lesions mentioned above. 

The biggest advantage of using dogs as a model is that they develop similar retinal morphological lesions as compared with human. Routine *in vivo* vasculature assessments, however, were impeded owing to the spontaneous diabetic cataract, particularly in the galactose-fed model; additional lensectomy is necessary [219, 223, 224]. Moreover, high maintenance cost, long-term follow-up period, and lack of molecular reagents, such as antibodies, make this model less commonly used for studying DR.

### 2.6. Swine Model of DR

Pig eye has become a useful tool in eye research because of its close similarities in the size as well as the basic retinal structure and vasculature to the human eye [226]. A number of models have been generated in order to study the retinopathy in swine upon diabetes, which include alloxan- and STZ-induced type 1 diabetic models. There is also a recently developed model of proliferative vitreoretinopathy that involved surgical procedures and intravitreal injection of retinal pigment epithelial (RPE) cells.

#### 2.6.1. Pharmacologically Induced Swine Models of DR

There are only limited reports on the retinal morphology of the chemically induced diabetic pigs. Instead, researchers make use of the large amount of specific retinal cells and vitreous available in the pig eyes for *in vitro* experiments [227, 228]. Nevertheless, reactivation of Müller cells was evident from the increased GFAP immunoreactivity from the ONL extending to the outer limiting membrane [227] in 2 to 3 months after onset of alloxan-induced diabetes. At around 4 months of hyperglycemia, pericyte degeneration in parallel to reduced the total number of BRB capillaries and capillary collapse were also observed [229]. Retinal vascular lesions, such as basement membrane thickening [230, 231] and rarefaction [231], were reported in pigs after 18 weeks of STZ-induced diabetes. Development of hyperglycemic cataract was also reported in this animal after 32 weeks of hyperglycemia that constrain the visualization of the vasculatures, such as fundus angiography [231].

#### 2.6.2. Swine Model for Angiogenesis Study

Recently, a new swine model of proliferative vitreoretinopathy has been described [232]. In brief, vitreal and retinal detachments were initially induced by vitrectomy and injection of subretinal fluid, respectively, prior to injection of cultured RPE cells into the vitreous cavity. Formation of contractile membranes on the inner retinal surface as well as localized tractional retinal detachments was evident and maintained after 14 days of induction while the retina reattached in the control animals at 3 days after the surgery. Further characterization of this model needs to be carried out before its use in therapeutic drug screening.

Although pig is a valuable model for disease study in human, high maintenance cost, requirement of special housing facilities, and lack of biochemical reagents make this model less commonly being used.

### 2.7. Monkey Model of DR

Monkey, a nonhuman primate, is considered to be a potential model in eye research owing to its structural similarity to human and, in particular, the presence of macula. The studies of DR in monkey can be divided into 3 groups: type 1 diabetic model, type 2 diabetic model, and model of VEGF-induced neovascularization. 

#### 2.7.1. Type 1 Diabetic Monkey Models

In an attempt to produce DR in monkeys, monkeys with type 1 diabetes that developed spontaneously as well as that resulted from total pancreatectomy or STZ injection were being used [233]. Unexpectedly, 37 out of 39 of these monkeys did not show any significant DR within 5 years of hyperglycemia. Animals with hyperglycemia of 6 to 15 years only showed mild disruption of the blood-retinal barrier. On the other hand, spontaneous or pharmacological induction of hypertension in the hyperglycemic monkeys, either by STZ injection or with spontaneous diabetes, resulted in ischemic retinopathies, such as cotton-wool spots which were found in the peripapillary region, microaneurysms, capillary dropout, capillary dilatation, focal intraretinal capillary leakage spots, arteriolar and venular occlusions, and atrophic macula, between 6 and 15 years of diabetes. The authors suggested that the fluctuating blood glucose levels and systemic blood pressure, but not hyperglycemic alone, play a role in the pathogenesis of DR.

#### 2.7.2. Type 2 Diabetic Monkey Models

DR studies have also been carried out in monkeys that spontaneously develop type 2 diabetes. While moderate retinal lesions can be identified in a case of monkey with 3 years of diabetic history, no detectable retinopathy was reported in a monkey with 15 years of diabetes [234]. The presence of these lesions was variable in individual animals, making it hard to deduce the precise onset of symptoms based on the diabetic duration. Among those showing retinopathies, cotton-wool spots, intraretinal hemorrhages, and nonperfused areas were the early observations. Progressive lesions, such as growing nonperfused area, which are associated with the formation small IRMAs and microaneurysms, as well as macular edema were also evident. Similar observations were also reported in another case study in which the subject is a monkey with at least 5 years of diabetes [235]. The authors have mentioned other histological abnormalities, including reduction of the thickness of the ONL and the inner and outer segments of the photoreceptor layers. Functional lesions were suggested by a loss in the amplitudes in the multifocal ERG, and they were virtually correlated to the nonperfused areas. Progressive reduction of amplitudes and delayed a-waves were also observed in the dark-adapted Ganzfeld ERG, suggesting a loss of function in the both inner and outer retina and reduced sensitivity in the photoreceptors, respectively. Moreover, it is reported that the occurrence of retinopathy is correlated with hypertension [234], which is coincidently similar to the descriptions in the type 1 diabetic monkey model.

#### 2.7.3. Monkey Models for Angiogenesis Study

VEGF-induced proliferative retinopathy has also been carried out in nonhuman primates [213]. In brief, a pellet containing human recombinant VEGF was implanted into the vitreous cavity of the animal. At 2 weeks after the implementation, severe BRB breakdown was noted. Retinal vascular dilation and tortuosity were further observed in 3 weeks. The abnormalities peaked at 3 weeks and regressed afterwards. Neovascularization was not detected in this model.

There are many limitations in using monkeys in DR studies. Apart from the variations in the onset of morphological abnormalities and the absence of advanced retinopathies, low birth rate, high cost, long duration of study, and the heightened ethical concern make this model unfeasible for the purpose of drug screening.

### 2.8. Zebrafish Model of DR

Despite the enormous ethical concern in the laboratory use of the mammals mentioned above, in particular the primates, the recent emergence of zebrafish can resolve this complication. Zebrafish is extensively used in the study of visual development and impairments owing to its similarity to those seen in human [236]. The distinctive pattern of the mammalian retinal cell layers, ranging from ganglion cell layer to retinal pigment epithelium, is observed in zebrafish [236]. The retinal vasculature is rather complex in adult zebrafish [237]. Blood supply to the retina is supported by the optic artery, which branches into four to nine major blood vessels. These vessels further arborize into smaller vessels towards the peripheral of the retina where anastomosis between the neighboring capillaries is present. This radial vascular network covers the entirely inner surface retina with direct contact with the GCL. Oxygen-deprived blood is collected in the circumferential vein surrounding the retina where the cilliary marginal zone is. DR can be studied in zebrafish via direct elevation of glucose in the surrounding as well as angiogenesis without the involvement of glucose.

#### 2.8.1. Glucose-Induced Diabetic Model of DR

In brief, zebrafish was exposed to freshwater with alternation between 2% and 0% glucose in every 24 hours for 30 days [238]. Hyperglycemia can be achieved in the animal in 1 day of immersion in the 2% glucose freshwater and maintained for at least 30 days with repeated hyperglycemic spikes every time after the removal from the glucose-freshwater. After 28 days of persistent hyperglycemia, the thickness of the IPL was significantly decreased, and yet no other abnormality has been observed [238]. The mechanism of glucose uptake in zebrafish is regulated by osmoregulation, in which influx of water, together with glucose, goes into their body as a result of high internal salt concentration. It has been reported that teleosts also have endocrine islet tissue containing hormone-producing cells which converge in the fish body, and the secretory teleost insulin is functional and is homologous to the human insulin [239]. This further validates the potential use of glucose-induced diabetic zebrafish in studying the retinopathy. Yet, morphological studies associated with the retinal vessels remained to be performed.

#### 2.8.2. Zebrafish Models for Angiogenesis Study

Two models to study angiogenesis in zebrafish are described below, namely, environmentally and transgenic-induced models. 

Retinal neovascularization can be achieved by keeping the zebrafish in hypoxic aquaria where the air saturation is gradually reduced to 10% (820 bbp) over a course of 48 to 72 hours and maintained for 12 days [240, 241]. In these studies, *fli*-EGFP-Tg zebrafish, which is a transgenic line that overexpresses EGFP in the vascular endothelium, was used for easy visualizing and imaging of the blood vessels. After 12 days of hypoxic challenge, neovascularization was observed in the retina evident by increased number of branch points, sprouts, and vascular area as well as reduced intercapillary distance [240]. This model can be useful for studying the development of angiogenesis or possibly for screening antiangiogenic pharmacological agents.

Retinal angiogenesis can also be induced in zebrafish via transgenic approach. Zebrafish carrying *vhl* ^−/−^ mutation displays an upregulation of hypoxia-inducible factor, which in turns triggers VEGF production and expression of the VEGF receptors [242]. By 5.75 days after fertilization (dpf), increased hyaloids and choroidal vascular networks were observed, followed by vascular leakage at 7.25 dpf. Excessive blood vessels were evident in the IPL, together with severe macular edema and retinal detachment at 7.5 dpf. However, this model is not commercially available, which limits its use in the field even though severe neovascularization and proliferative retinopathy are observed.

Zebrafish is very small in size; therefore, its maintenance is simple, convenient, and inexpensive. They have a short life span and a large breeding size, which in turn allow a shorter experimental turnover time. Moreover, a number of studies showed that genes of interest can be specifically induced, deleted, or overexpressed in zebrafish, allowing mechanistic studies of diseases [243]. As a consequence, researchers have developed certain zebrafish models in order to study DR, including glucose-induced diabetic model and models specifically of angiogenesis. However, the retinal cells layers differ in thickness and thereby the number of cells, the findings may under- or overestimate the contribution of a specific cell type in regard to the pathogenesis of DR. In terms of the vasculature in zebrafish, the growth of the tertiary plexus of blood vessels in the INL is absent and the venous system is different from those in human. Therefore, researchers should be aware that using zebrafish may lead to potential discrepancy in cellular and vascular aspects and may not truly reflect the pathological development of DR in patients. Moreover, owing to the limited supply of tissue from a single animal, skillful techniques and a large quantity of eyeballs are required in dissection and for molecular analysis.

A summary of the temporal morphological and functional lesions of the animal models, other than rodents, described above is shown in Table 4.  Table 5 displays general comparisons of the use of different animals in studying DR.

## 3. Conclusions

Animal models are very important in understanding the pathogenesis of diseases in human, defining novel therapeutic targets as well as screening of novel therapeutic drugs. In this review, a number of animal models of DR have been described and compared, ranging from different species to different induction methods of diabetes or angiogenesis, together with their corresponding temporal morphological and functional lesions. Up to date, there is no single model which can mimic the development of DR as in human, that is, from the very early cellular and vascular abnormalities to the proliferative stage, and subsequently retinal detachment, as a result of prolonged hyperglycemia. Rodents have been extensively used in DR studies owing to their small size and the ability to develop retinopathies within a relatively short period of time. The availability of a collection of transgenic mice further aids in elucidating the role of target molecules in DR. Since the STZ-induced diabetic rodents are the most frequently used models in studying the associated retinopathy, we have summarized the administration dosage and paradigm published in the recent years as a reference to other researchers. Nevertheless, a majority of the diabetic rodent models only demonstrated the early symptoms of DR, which restricts their applications in mechanistic studies and drugs screening targeting the early progression of DR. Some higher-order animals showed relatively advanced retinopathies, such as neovascularization, upon induction of diabetes, yet they still cannot imitate the later stage of DR as seen in human. Moreover, high maintenance cost, long duration of study, and lack of molecular reagents, such as antibodies, as well as ethical concern further limit their use in studies. Zebrafish is another model that emerged recently in studies of DR; however, further characterization needs to be done. The presence of neovascularization is controversial in some animal models; such variation may come from animals of different strain and age, individual variation, and/or even the detection methods. Therefore, we suggested that researchers should have a bigger sample size, use at least two detection methods, such as fluorescence angiography, immunohistochemical staining of blood vessel marker on retinal flat mounts or cross-sections in combination with molecular analysis, in order to have a more convincing claim. Furthermore, overexposure of the fluorescence staining and “cleanness” of the section, particularly in the flat mounts, are other issues that researchers should be aware of. Although neovascularization can be observed in animals overexpressing VEGF, either via transgenic approach or direct introduction, the development of neovascularization is not caused by prolonged hyperglycemia. Therefore, using these models in studies of the etiology of the disease or the development of preretinal neovascularization should be avoided. Another approach for induction of neovascularization is by hypoxic challenge in rodents; however, regression was reported within a few days, which may limit the duration of the drug treatment versus the formation of new vessels. As outlined in this review, individual model of DR has different strengths and weaknesses; careful consideration should be made in choosing appropriate models to address the research questions. 

## Figures and Tables

**Table 1:** Existing methods for induction of diabetes in rodents using STZ.

	Compensation of insulin	Dosage	Animal strain, age or weight	Onset of hyperglycemia
*Mouse *				
Single dose	No	150 mg/kg, i.p.	C57Bl/6, 8–10 wk old	6 wk* [53]
	No	150–180 mg/kg, i.p.	C57Bl/6, 10–12 wk old	2 wk [54]
	No	180 mg/kg, i.p.	C57Bl/6, 10 wk old	1 day [41, 55]
	No	200 mg/kg, i.p.	C57Bl/6J, 10 wk old [56]; F1 hybrid (BALBc/129Sv × C57Bl/6J), 16 wk old [43]	3 days [56]
	Yes	180 mg/kg, i.p.	C57Bl/6, 9–12 wk old	1 wk [45, 48, 57, 58]
	Yes	200 mg/kg, i.p.	C57Bl/6, 12 wk old	1 wk [47]
Multiple dose	No	40 mg/kg, i.p., for 5 consecutive days	C57Bl/6, 10 wk old	1 wk [59]
	No	45 mg/kg, i.p., for 5 consecutive days	C57Bl/6, 7-8 wk old	3 days [60]
	No	50 mg/kg, i.p., for 5 consecutive days	C57Bl/6J, 4-5 wk old [61]; C57Bl/6J, 8-9 wk old [46, 62]; C57Bl/6 [63]	1 wk [46]
	No	55 mg/kg, i.p., for 5 consecutive days	C57Bl/6, 8–10 wk old [42, 64–66]	1 wk [42, 64, 66]; 2 wk [65]
	No	60 mg/kg, i.p., for 3 consecutive days	C57Bl/6, 5–8 wk old [34, 49, 50]	1 wk [34, 49, 50]
	No	60 mg/kg, i.p., for 5 consecutive days	C57Bl/6, 6 wk old [40]; C57Bl/6J, 22 g [44]; C57Bl/6, 22 wk old [67]	1 wk [40, 44]; 2 wk [67]
	No	75 mg/kg, i.p., for 3 consecutive days	C57Bl/6, 3–5 months old	5 wk* [68]
	No	80 mg/kg, i.p., for 3 consecutive days	C57Bl/6, 6–8 wk	1 wk [69]
	No	80 mg/kg, i.p., for 5 consecutive days	F1 hybrid mice (FVB/N × C57Bl/6J), 10 wk old	1 wk [33]
	No	100 mg/kg, i.p., for 2 consecutive days	C57Bl/6J, 6–8 wk old	1 wk [70]
	Yes	50 mg/kg, i.p., for 3 alternate days	C57Bl/6, 23–36 g	4 wk* [71]
	Yes	50 mg/kg, i.p., for 5 consecutive days	C57Bl/6 [72]	Did not mentioned
	Yes	60 mg/kg, i.p., for 5 consecutive days	C57Bl/6 [73]; C57Bl/6J [38]	1-2 wk [38, 73]

*Rat *				
Single dose	No	30 mg/kg, i.v.	Wistar rats, 6 wk old	3 wks [74]
	No	45 mg/kg, i.p.	Sprague-Dawley (SD) rats, 200–250 g [75, 76]; Wistar rats, 220–250 g [77]	1 day [77]; 3 days [76]; 1 wk [75]
	No	45 mg/kg, i.v.	Wistar rats, 130–150 g	1 day [78]
	No	50 mg/kg, i.p.	Brown Norway (BN) rats, 8 wk old	2 days [79]
	No	55 mg/kg, i.p.	Wistar rats, 8–12 wk old [80, 81]	3 days [80, 81]
	No	60 mg/kg	Lewis rats [82]	Did not mentioned
	No	60 mg/kg, i.p.	BN rats, 200–250 g [83]; SD rats, 6–8 wk old [84–91]; SD rats, 230–350 g [92, 93]; SD rats [94]; Wistar rats, 140–200 g [95–97]; Wistar rats, 200–300 g [98–100]; Long-Evens (LE) rats, 250–300 g [101]	1 day [83, 89]; 2 days [86–88, 91, 100]; 3 days [84, 85, 92, 93, 97, 98] 1 wk [90, 96]
	No	65 mg/kg, i.p.	SD rats, 150–200 g [102–106]; SD rats, 240–275 g [107, 108]; Wistar rats, 8-9 wk old [109–111]	1 day [106]; 2 days [107, 109–111]; 3 days [102, 103]; 1 wk [104, 105]
	No	65 mg/kg, i.v.	BN rats, 150–200 g [112]; LE rats, 150–200 g [112]; Wistar rats, 160–170 g [113, 114]	3 days [112]; 2 wk* [113, 114]
	No	70 mg/kg, i.p.	SD rats, 8 wk old	12 wk* [115]
	No	75 mg/kg, i.p.	LE rats, 200–250 g	2 days [116]
	No	80 mg/kg, i.p.	LE rats, 6–8 wk old	1 wk [69]
	Yes	45 mg/kg, i.v.	Wistar rats, 12 wk old	3 days [117]
	Yes	50 mg/kg, i.p.	SD rats, 6 wk old	2 wk [118]
	Yes	55 mg/kg, i.p.	SD rats, 200 g	2-3 days [119]
	Yes	55 mg/kg	Lewis rats, 200 g [120, 121]; SD rats, 200 g [120]; Wistar rats, 200 g [120]	2 wk* [120, 121]
	Yes	60 mg/kg, i.p.	Wistar rats, 129–170 g [58] Wistar rats, 240–280 g** [122]	1 day [122]; 1 wk [58]
	Yes	65 mg/kg, i.p.	SD rats, 260 g	4 wk* [123]
	Yes	65 mg/kg, i.v.	SD rats, 200 g	4 wk* [124]
	Yes	70 mg/kg, i.p.	LE rats, 8 wk old	3 days [125, 126]
	Yes**	80 mg/kg, i.p.	SD rats, 200 g	1 wk [127]

*Indicates the earliest time point being mentioned by the authors.

**Comparison between the insulin-treated versus untreated animals.

**Table 2:** Comparison of the morphological and functional lesions in mouse models of DR.

Animal model	Type of diabetes	Onset of hyperglycemia	Temporal morphological lesions in retina upon development of hyperglycemia		Temporal functional lesions in retina upon development of hyperglycemia (ERG, SLO, fMRI)
			Cellular	Vascular	
STZ injection	Type 1	Within 1 wk of injection	4 wk: astrocyte activation [32] 5 wk: (i) Increased astrocyte number [33] (ii) Reactive gliosis [33] 6 wk: apoptosis of RGCs [35] 6–14 wk: reduced RGCs [34, 35]* 10 wk: reduced thickness of INL and ONL [35] 6–9 mth: pericytes loss [32, 37]	8 days: upregulation of vascular permeability [41] 4 wk: (i) Decreased retinal arteriolar and venular RBC velocity [45, 46] (ii) Decreased retinal arteriolar and venular blood flow rate [45, 46] (iii) Decreased arterial velocity [47] (iv) Decreased arteriolar and venular diameters [48] 8 wk: (i) Vessel leakage [42] (ii) Increased leukocyte number [40] (iii) Leukostasis [38] 17 wk: (i) Capillary basal laminar thickening [43] (ii) Neovascularization [44] 6 mth: apoptosis of vascular cells [32] 6–9 mth: acellular capillaries [32, 37]	4 wk: (i) Decreased OP3 and ΣOPs in ERG [49, 50] (ii) Prolonged implicit time of OP2-3 in ERG [50] 7 wk: decreased pattern of ERG amplitude [51] 6 mth: decreased a-wave and b-wave amplitudes [37]

Alloxan injection	Type 1	Within 4 days of injection	3 mth: shortening of dendrites of microglial cells [128]		3 wk: decreased b-wave in ERG [129, 130] 3 mth: (i) Decreased b/a wave amplitude ratio in ERG [128] (ii) Delayed OPs in ERG [128]

Galactose-fed	—	—	16–22 mth: decreased endothelial cell [132, 133] 20–26 mth: pericyte loss [131, 133]	15–21 mth: acellular capillaries [131–133] 21 mth: saccular microaneurysms [131] 21-22 mth: capillary basal laminar thickening [131, 132]	

Ins2^Akita^	Type 1	4 wk of age	8 wk: reactivated microglia [134] 12 wk: (i) Increased apoptosis [136] (ii) Reduced RGCs in the peripheral retina [135] (iii) Changes in dendrite of ON-type RGCs [135] (iv) Abnormal swelling on somas, axons, and dendrites in RGCs [135] 22 wk: (i) Change in astrocyte morphology and less contact with the vessels [134] (ii) Reduced thickness of IPL and INL [134] (iii) Reduced RGCs [134]* 6 mth: decreased cholinergic and dopaminergic amacrine cells [136]	8 wk: increased leukocyte number [134] 12 wk: increased retinal vascular permeability [134] 26 wk: decreased arteriolar and venular RBC velocity, shear rate, and flow rate [138] 31–36 wk: increased acellular capillaries [134] 8 mth: (i) Retinal neovascularization [137] (ii) Formation of new capillary beds [137] (iii) Formation of new blood vessels in OPL [137]	8 mth: decreased a-wave and b-wave amplitudes in ERG [137]

NOD	Type 1	12–30 wk of age	4 wk: apoptosis of pericytes, endothelial cells, and RGCs [140]	4 wk: (i) Capillary basement membrane thickening [140] (ii) Perivascular edema [140] 4 mth: (i) Vasoconstriction or degeneration in some of the major vessels [141] (ii) Poorly defined microvessels [141] (iii) Disordered focal proliferation of new vessels [141]	

db/db	Type 2	8 wks of age	6 wk: (i) Reduced RGC number [144] (ii) Reduced thickness of the central total retina, INL, and photoreceptor layers [144] 18 wk: pericyte loss [145] 13 mth: glial reactivation [146]	14 wk: basement membrane thickening [147] 26 wk: acellular capillaries [145] 13 mth: (i) Vessel leakage [146] (ii) Increased vessel density in the INL [146]	

KKA^y^	Type 2	6–8 wk of age	4 wk: increased neuroretinal apoptotic cells in RGC layer [149] 3 mth: increased neuroretinal apoptotic cells in the INL [149]	3 mth: basement membrane thickening [149]	

OIR	—	—	P18: (i) Reduced thickness of total retinal [153] (ii) Reduced thickness of IPL [153] (iii) Absence of the deep plexus [153] (iv) Reduced outer segment length [153] (v) Microglial reactivation [153] (vi) Increased gliotic Müller cells and microglia [153]	P18: (i) Aberrant intravitreal neovascularization across all eccentricities [153] (ii) Reduced vessels in the deep plexus [153] (iii) Reduced vessels in the inner retinal plexus [153]	P18: (i) Reduced a-wave and b-wave amplitudes in ERG [153] (ii) Delayed b-wave implicit time in ERG [153] (iii) Reduced OP3 and OP4 in ERG [153]

Kimba	—	—	9 wk of age: pericyte loss [158]	P28 of age: (i) Microaneurysms [157] (ii) Vascular leakage [157], but stops at 9 wk of age [158] (iii) Capillary blockage, dropout, and hemorrhage [157] 6 wk of age: (i) Leukostasis in veins and capillaries [158] (ii) Acellular capillaries [158] 9 wk of age: (i) Reduced vessel covered area [158] (ii) Reduced vessel length [158] (iii) Reduced crossing points [158] (iv) Vessel tortuosity [158]	

Akimba	Type 1	4 wk of age	8 wk of age: (i) Uneven thickness in the retina in OCT [159] (ii) Reduced photoreceptor layer thickness [159] (iii) Retinal edema [159] (iv) Retinal detachment [159] 24 wk of age: reduced RGCs number and neural retinal thickness [159]	8 wk of age: (i) Capillary dropout [159] (ii) Microaneurysm [159] (iii) Hemorrhage [159] (iv) Vascular leakage, stops by 20 wk of age [159] (v) Vascular dilatation [159] (vi) Venous loops [159] (vii) Vessel tortuosity [159] (viii) Vessel beading [159] (ix) Neovascularization [159]	

*Some studies reported absence of reduction of RGCs [36–39].

The observations reported at a particular time point, which was chosen by the authors, may not totally reflect the sequential processes.

**Table 3:** Comparison of the cellular and functional lesions in rat models of DR.

Animal model	Type of diabetes	Onset of hyperglycemia	Temporal morphological lesions in retina upon development of hyperglycemia		Temporal functional lesions in retina upon development of hyperglycemia (ERG, SLO, fMRI)
			Cellular	Vascular	
STZ injection	Type 1	Within 1 wk of injection	2 wk: increased apoptotic cells [136] 1 mth: (i) Microglial cells hypertrophy [160] (ii) Increased Müller cells and microglia cell number [160, 161] (iii) Reduced astrocyte number [161] (iv) Apoptosis of a few photoreceptor cells [162] 6 wk: (i) Reduced astrocyte number [118] (ii) Reduced astrocyte processes [118] (iii) Reduced NeuN-positive ganglion cells in GCL [160] (iv) Reduced cells in the ONL [163] (v) Reduced total retinal thickness [163] 4 mth: reduced NeuN-positive neurons in INL [160]	2 wk: BRB breakdown [161, 163] 8 wk: increased adherent leukocytes [164] 12 mth: arterial or venous capillaries basement membrane thickening [165]	2 wk: reduced b-wave amplitudes in ERG [166] 4 wk: decreased positive scotopic threshold response in ERG [167] 8 wk: decreased OPs in ERG [167] 10 wk: reduced a-wave amplitudes in ERG [166] 11 wk: (i) Reduced a-wave implicit time in ERG [168] (ii) Delayed OPs in ERG [168]

Alloxan injection	Type 1	Within 1 wk of injection	12 mth: pericyte loss [169]	2 mth: (i) Neovascularization limited to the midperipheryregion[170] (ii) Extravascular accumulation of monocytes and granulocytes [170] 5 mth: (i) Neovascularization in the midperipheryregionand the center [170] (ii) Capillary endothelial cells swelling [170] 8 mth: retinal microvascular cell death [171] 9 mth: neovascularization in all regions [170] 12 mth: (i) Acellular capillaries [169] (ii) Basement membrane thickening [169]	

Galactose-fed	—	—	12 mth: pericyte loss [169] 28 mth: (i) Gliosis [172] (ii) Disruption of retinal layers [172]	6 mth: retinal microvascular cell death [171] 12 mth: (i) Acellular capillaries [169] (ii) Basement membrane thickening [169] 28 mth: (i) Capillary dilation [172] (ii) Microaneurysm formation in the IPL and the INL [172]	

BB	Type 1	3-4 mth of age	4 mth: absence of infolding or derangement of the basal plasmalemma of the RPE [173] 8 mth: (i) Reduced pericyte number [174] (ii) Reduced pericyte to endothelial cell ratio [174]	2 mth: capillary dilation [175] 4 mth: basement membrane thickening [175] 8 mth: microinfarctions with areas of nonperfusion [175]	

WBN/Kob	Type 2	9–12 mth of age	5 mth of age (prediabetic): reduced thickness of outer segments and ONL [176] 2 mth: (i) Reduced thickness of total retinal layer, OPL [176] (ii) Visual cells disappeared [176]	1 mth: capillaries clustered into small tortuous knots [177] 5 mth: capillary basement membrane thickening [176] 6 mth: (i) Increased capillary loop [177] (ii) Reduced number of capillary [177] 12 mth: (i) Increased fibrovascular element proliferation in the vitreous [178] (ii) Increased intraretinal neovascularization [178] (iii) Increased hyalinization of intraretinal vessels [178]	

ZDF	Type 2	6-7 wk of age	6 mth: (i) Increased apoptotic pericytes [179] (ii) Increased pericyte ghosts [179]	5 mth: (i) Capillary basement membrane thickening [180, 181] (ii) Increased capillary cell nuclear density [180, 181] 6 mth: (i) Increased apoptotic endothelial cells [179] (ii) Acellular capillaries [179]	

OLETF	Type 2	5 mth of age	9 mth: (i) Damaged endothelial cells [182] (ii) Reduced ratio of pericyte area to the total capillary cross-sectional area [183] 14 mth: (i) Decreased thickness of INL [182] (ii) Decreased thickness of photoreceptor layer [182] (iii) RPE decreased in height [182] (iv) Basal infoldings were poorly developed [182]	6 wk: leukostasis [184] 9 mth: (i) Capillary basement membrane thickening [182, 183] (ii) Caliber irregularity [183, 185] (iii) Narrowing of arteries [185] 9–12 mth: (i) Tortuosity [182, 183, 185] (ii) Microaneurysms [182, 183, 185] (iii) Loop formation in capillary [182, 183, 185]	

GK	Type 2, nonobese	4–6 wk of age	7 mth: increased endothelial/pericyte ratio [186]	1 mth: (i) Reduced retinal segmental blood flows [186] (ii) Increased retinal mean circulation time [186] 3 mth: increased BRB permeability [187]	

SDT	Type 2, nonobese	5 mth of age	20 wk: increased apoptotic cells in the GCL and the INL [188] 48 wk: distortion of retina and protruded optic disc [189] 50 wk: tractional retinal detachment with fibrous proliferation [190] 15 mth: (i) Proliferative retinopathy without vascular nonperfusion (53% of rats) [191] (ii) Traction retinal folds [191]	24 wk: leukostasis [192] 48 wk: leakage of fluorescein around the optic disc [189]	4 wk: delayed peak latency of the ΣOPs in ERG [189] 24 wk: (i) Reduced a-wave, b-wave, and OPs amplitudes in ERG [193] (ii) Prolonged implicit times in a-wave, b-wave, and OPs in ERG [193]

OIR	—	—	P18: (i) Reduced thickness in INL, IPL, and total retinal layer [194] (ii) Müller cell gliosis in peripheral region [194] (iii) Reduced astrocyte number [195] (iv) Impaired pericyte endothelial interactions [195] (v) Disorganized and thinning of the outer segment [196]	P18: (i) Intravitreal neovascularization [195] (ii) Underdevelopment of the outer vascular plexus [195] (iii) Increased tortuosity in arterioles [197]	P16: reduced a-wave and b-wave amplitudes in ERG [197]

The observations reported at a particular time point, which was chosen by the authors, may not totally reflect the sequential processes.

**Table 4:** Comparison of the morphological and functional lesions in models of DR other than rodents.

Animal model	Type of diabetes	Onset of hyperglycemia	Temporal morphological lesions in retina upon development of hyperglycemia		Temporal functional lesions in retina upon development of hyperglycemia (ERG, SLO, fMRI)
			Cellular	Vascular	
*Rabbit *					
STZ injection	Type 1	Did not mentioned	19 wk: (i) Moderate vasculopathy with hard or soft exudates, widespread hemorrhages in 10% of eyes [211] (ii) Serious vasculopathy with serious retinal and preretinal hemorrhages, vascular lesions, hemovitreous, and venous thrombosis in 40% of eyes [211] (iii) Proliferant retinopathy in 50% of eyes [211]		
Diet-induced	Type 2	Between 12 and 24 wk of diet		12 wk of special diet: (i) Increased microaneurysms [212] (ii) Presence of hyperfluorescent dots consistent with microaneurysms [212]	
VEGF implant	—	—		7 days after implementation: increased dilation and tortuosity of retinal vessels [213] 14 days after implementation: (i) Presence of numerous small tortuous blood vessels [213] (ii) Leakage of blood vessels [213] 21 days after implementation: neovascularization [213]	

*Cat *					
Pancreatectomy	Type 1	1-2 wk after surgery		3–10 mth: basement membrane thickening [218] 5 yr: microaneurysms [216] 6.5 yr: small intraretinal hemorrhages [216] 7.5 yr: (i) Presence of area of capillary nonperfusion [216] (ii) IRMA [216] 8.5 yr: small foci of neovascularization [216]	

*Dogs *					
Galactose-fed	—	—	19 mth of feeding: pericyte loss [222] 24 mth of feeding: uneven distribution of endothelial cells [222] 60 mth of feeding: (i) Soft exudates [222] (ii) Gliosis in the nerve fiber layer [222]	27-28 mth of feeding: microaneurysm formation [222, 223] 33 mth of feeding: (i) Dot and blot hemorrhages [222] (ii) Degenerated microaneurysms and varicose enlargements [222] 36 mth of feeding: (i) Confluent hemorrhages [222] (ii) Increased acellular capillaries [225] (iii) Increased endothelial cells to pericytes ratio [225] 37–46 mth of feeding: nonperfusion [223, 224] 56 mth of feeding: (i) Preretinal hemorrhage [222] (ii) Broad areas of nonperfusion [222] (iii) IRMA [222] 60 mth of feeding: (i) Occluded arterioles [222] (ii) Large arteriovenous shunts [222] (iii) Node formation on arterial and arteriolar walls [222] 66 mth of feeding: (i) Intravitreal hemorrhage [222] (ii) Partial posterior vitreous detachment [222] 68 mth of feeding: neovascularization [224] 84 mth of feeding: intravitreal retinal-vascular growth [222]	
STZ/alloxan injection			4 yr: (i) Pericytes loss [220] (ii) Loss of smooth muscle cells [220]	3 yr: basement membrane thickening [220]	

*Pigs *					
Alloxan injection	Type 1	15 days	90 days: Müller cells reactivation [227] 20 wk: pericyte loss [229]	20 wk: (i) Reduced total number of BRB capillaries [229] (ii) Capillary collapse [229]	
STZ injection	Type 1	1 wk		18 wk: (i) Basement membrane thickening [230, 231] (ii) Rarefaction [231]	
Surgery + RPE injection	—	—	14 days: (i) Formation of contractile membranes on the inner retinal surface [232] (ii) Localized traction retinal detachments [232]		

*Monkey *					
Spontaneous, STZ-induced, or pancreatectomy	Type 1	—	6–15 yr: atrophic macula [233]	6–15 yr: (i) Cotton-wool spots in the peripapillary region [233] (ii) Microaneurysms [233] (iii) Capillary dropout [233] (iv) Capillary dilatation [233] (v) Focal intraretinal capillary leakage spots [233] (vi) Arteriolar and venular occlusions [233]	
Spontaneous obese	Type 2	—	3–8 yr: (i) Macular edema [234] (ii) Reduction thickness of ONL [235] (iii) Reduction thickness of inner and outer segments of the photoreceptor layers [235]	3–8 yr: (i) Cotton-wool spots [234] (ii) Intraretinal hemorrhages [234] (iii) Nonperfused areas [234] (iv) Small IRMAs [234] (v) Microaneurysms [234]	5 yr: (i) Amplitude loss in multifocal ERG [235] (ii) Reduced amplitudes in ERG [235] (iii) Delayed a-waves in ERG [235]
VEGF implant	—	—		2 wk after implementation: severe BRB breakdown [213] 3 wk after implementation: retinal vascular dilation and tortuosity [213]	

*Zebrafish *					
Glucose-induced	Type 1	1 day	28 d: decreased IPL thickness [238]		
Hypoxia-induced	—	—		12 days: (i) Increased branch points [240] (ii) Increased number of sprouts [240] (iii) Reduced intercapillary distance [240] (iv) Increased vascular area [240]	
*Vhl* ^−/−^	—	—		5.75 dpf: increased hyaloid and choroidal vascular networks [242] 7.25 dpf: vascular leakage [242] 7.5 dpf: (i) Presence of blood vessels in the IPL [242] (ii) Severe macular edema [242] (iii) Retinal detachment [242]	

The observations reported at a particular time point, which was chosen by the authors, may not totally reflect the sequential processes.

**Table 5:** Comparison of the strengths and weaknesses of different animal models of DR.

Animal	Strength	Weakness
Mouse	(i) Cellular lesions are extensively studied (ii) Availablility of many transgenic models, which allows the study of the role of particular genes in the development and pathophysiological progression of DR (iii) Duration of developing early DR symptoms is short (i.e., within weeks to months) (iv) Small in size, easy to handle and house (v) A wide range of molecular reagents are available	(i) Only early DR lesions were observed (ii) PDR does not spontaneously occur and it can only be induced with the aid of models of angiogenesis (e.g., OIR, occlusion, and VEGF-induced models)

Rat	(i) Cellular lesions are extensively studied (ii) Duration of developing early DR symptoms is short (i.e., within weeks to months) (iii) Small to medium in size, relatively easy to handle and house (iv) A wide range of molecular reagents are available	(i) Only early DR lesions were observed, except in SDT rats (ii) PDR will not spontaneously occur and it can only be induced with the aid of models of angiogenesis (e.g., OIR, I/R models)

Higher-order mammals including rabbit, cat, dog, pig, and nonhuman primates	(i) Relatively similar to the pathophysiology of DR in human (ii) Larger tissues which allow easier *in vivo* examinations (iii) Routine sampling of body fluids is allowed	(i) Longer life span, require longer period of time to develop DR (ii) Large in size, difficult to handle and house (iii) Lack of reagents for molecular studies (iv) Heightened ethical concern

Zebrafish	(i) Genes of interest can be easily targeted induced, deleted, or overexpressed (ii) Maintenance is simple, convenient, and inexpensive (iii) Short life span and large breeding size, which allow shorter experimental turnover time (iv) Minimal ethical concern	(i) Thickness of retinal layers and retinal vasculature is different (ii) Retina is anatomically less similar to human (iii) Skillful technique is required due to small size of tissue (iv) Lack of reagents for molecular studies (v) Not being widely used
